# Copy number variation as a tool for implementing pregnancy as an aging model

**DOI:** 10.18632/aging.204936

**Published:** 2023-08-28

**Authors:** Mariana Andrawus, Lital Sharvit, Noga Touitou, Batia Lerrer, Haim Y. Cohen, Gil Atzmon

**Affiliations:** 1 Department of Human Biology, University of Haifa, Haifa 3498838, Israel; 2 Faculty of Life Sciences, Bar-Ilan University, Ramat-Gan 5290002, Israel

**Keywords:** aging, pregnancy, copy number variation, gene expression

## Abstract

Copy number variations (CNV) are a major contributor to genome variability and have been linked to aging and other degradable phenotypes such as pregnancy physiology. To demonstrate how pregnancy can be used as a model of aging, we used CNVs from pregnant mice. Candidate CNVs were selected by applying case-control analysis in human centenarians compared with control groups. These CNVs were aligned with the mouse genome and their copy variation was assessed using qRT-PCR in liver and blood tissue samples from pregnant mice throughout pregnancy (baseline; first, second, and third trimester; post-partum). Eight of the ten selected CNVs demonstrated a significant decline/increase trend throughout the pregnancy followed by opposite direction soon after delivery in the liver and blood of the mouse tissues. Furthermore, significant differential expression was detected among the candidate CNVs’ close vicinity genes (*APA2A, LSS, RBDHF1, PLAAT1*, and *SCL17A2*), but not in the *WSCD2* gene. Establishing a genetic link between longevity and pregnancy is a significant step toward implementing the pregnancy process as a model for aging. These results in pregnant mice highlight the mechanism and similarities between pregnancy and aging. Investigating the mechanisms that cause such rejuvenation after labor could change our aging treatment paradigm.

## INTRODUCTION

Aging is a complex multifactorial process of gradual physiological decline that affects tissue function and renders organism's frail and susceptible to disease and death over time. Aging can occur at different rates across different organisms, and even within the same species [1]. During aging, the ability of tissues to regenerate is reduced and healing recovery is delayed [2]. The efforts invested in studying the aging process are immense; however, due to its complexity and lengthy duration, the acquired knowledge is far from complete. This is partly due to the lack of an accurate translatable short-lived human model capable of simulating the aging process in a short period of time. The use of human premature aging diseases seems to be the solution to such a formidable challenge. However, while they provide a unique context for studying the aging process, their pathological manifestation obscures the typical aging mechanisms, forcing us to return to the drawing board. To overcome these obstacles, we propose pregnancy as a translatable, short-term, and natural new human model for aging which demonstrates several similar physiological and cellular degradation mechanisms [3].

Pregnancy is a distinctive and complex period in a woman’s life. During pregnancy, all body systems encounter serious challenges, including physiological, biological, and functional changes [4]. Additionally, significant alterations arise as a result of functional and structural adaptation during pregnancy. Likewise, pathological conditions such as hypertension, diabetes, dementia-like, and cardiovascular complications are common in pregnancy [3]. These aging-like pregnancy-related phenomena may serve as an effective model to reveal specific areas of future research to increase our understanding of the aging process. To further implement pregnancy as a model for aging, we assessed predisposed genetic variation (copy number variations, or CNVs) associated with aging in a pregnant animal model.

CNVs are structural variations of DNA that account for both natural genomic variability and risk for human diseases. CNVs account for an estimated 13 percent of the genome in one or more individuals, indicating that CNVs can be significant contributors to heritable variation [5]. CNVs have been extensively studied over the last decade in the context of physiological, pathological, and performance outcomes in human, mice, and other organisms [6, 7]. CNVs have recently gained prominence as a source of genetic diversity, and they are expected to play a role in functional variation by changing gene structure and quantity, varying gene regulation, and revealing recessive alleles [8].

Although it was suggested that the accumulation of somatic CNVs is a result of the aging process, predisposing cell types to cancer progression and neurological diseases, an alternative hypothesis suggests that this event is a natural, and even a critical part of cell proliferation. Some evidence has shown that CNVs are a natural feature of the mammalian placental genome and are controlled systematically throughout gestation [9].

Interpretation requires deep expertise in human development as well as ongoing research, and the study of CNV can highlight novel ways for prevention, diagnosis, and treatment [10–12].

The physiological processes and cellular senescence and degradation seen in pregnancy mimic mechanisms related to aging. Aging is defined by a linear degradation without recovery. While in pregnancy, there is an unusual rejuvenation that reverses the damage caused by stress to original levels postpartum. Hence, we assume that pregnant women will response different to these changes may, in turn, provide a useful clue for the aging process and age-related disease pathogenesis in a short-duration model (i.e., nine months). We hypothesize that pregnancy can serve as a model for aging by demonstrating similar biomarkers, pathologies, and genetic and epigenetic effects [3]. To test this hypothesis, we designed a study that assesses CNVs associated with human longevity (unpublished results) in pregnancy. We evaluate CNVs in blood samples collected from mice at baseline (before pregnancy), across pregnancy (first, second, and third trimesters), and following the delivery. We examine systematically the incidence and distribution of CNVs which could be causative for the specific changes that occur in aging and across gestation. Such knowledge on physiological decline throughout pregnancy and the remarkable rejuvenation right after delivery will assist with revolutionizing our approach to treating the elderly.

## MATERIALS AND METHODS

### Affymetrix microarray

Whole-genome profiling of CNVs in the centenarians’ and control blood samples was carried out using Affymetrix microarray-based CNV calling. Total DNA was extracted from 287 elderly and 230 controls and then hybridized to Affymetrix microarray GeneChip, which consists of 21,560 distinct CNV regions.

### Candidate CNV selection (Figure 1)

#### Mouse model

A total of seven female C57BL/J mice were including in this study. Approximately 150–200 μL of blood was collected from mice before during (1 tr, 2 tr, 3 tr), and after pregnancy. In addition, liver tissue from mice before, during, and after pregnancy was isolated. Subsections were snap frozen and stored at −80°C for further analysis.

**Figure 1:**
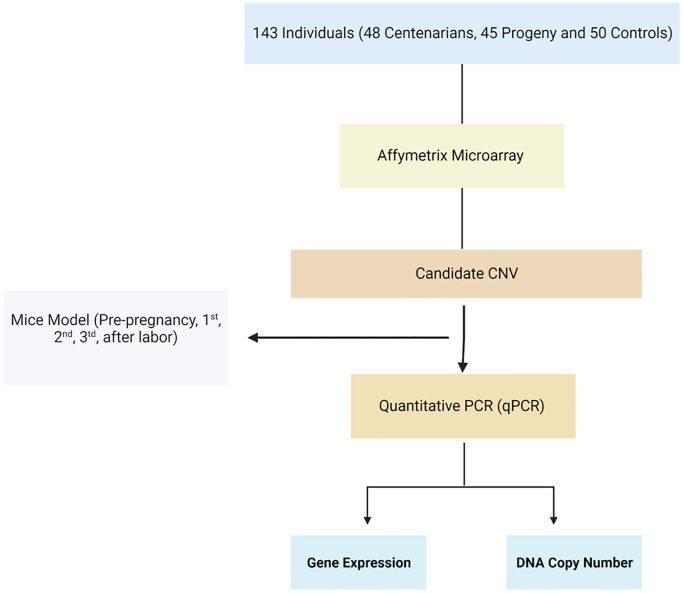
**Flowchart of the study.** Homolog candidate CNVs and their flanking genes were evaluated among mice during pregnancy using qRT-PCR.

### DNA extraction

DNA was extracted from mice blood samples and liver tissue following the manufacturer’s protocol using a high pure PCR template preparation kit (Roche, Penzberg, Germany). All DNA samples were tested for purity and integrity using a nanodrop (Implen, Munich, Germany) and DNA were stored at −20°C until use.

### RNA extraction

Total RNA was isolated using the TRIzol reagent (Bio-Tri reagent) protocol. In short, frozen tissue samples were weighed and processed on ice to prevent thawing. In preparation for RNA extraction, 5 mg of tissue was added to 1000 μl of the appropriate lysis buffer (i.e., TRIzol). Samples were homogenized using a homogenizer resting on ice. Homogenates were subjected to a single freeze–thaw cycle to aid cell lysis and then cleared by centrifugation at 12,000 × g for 10 minutes at 4°C. RNA extractions were performed according to the manufacturer’s instructions. RNA was eluted in 40 μl of RNase free water and stored at −80°C.

### qRT-PCR

RNA samples were treated with DNase I (DNA-free, Ambion, USA), and 1 μg was taken for first-strand cDNA synthesis (iScript, Bio-Rad, USA) in a 20-μl volume. Aliquots of 1 μl of cDNA were used for each real-time PCR reaction. qRT-PCR was used to confirm the CNVs detected by microarray analysis. Relative quantification of gene transcription was performed using Fast SYBR Green (Applied Biosystems, USA), and 1 μl of cDNA was generated from 50 ng total RNA; 5 μL of the cDNA was mixed with 15 μL of the reaction mixture containing 7.5 μL of the master mix, forward primers 0.5 μL, and reverse primers 0.5 μL. The sequence of primers used was designed using the Primer3+ web tool (http://bioinfo.ut.ee/primer3-0.4.0/) layout in the Supplementary Materials (Supplementary Tables 1, 2). For the CNV assessment, all reactions used 10 ng of DNA in a final volume of 10 μL containing 5 μL SYBER green Master Mix, 1 μL primers, 2 μl water, and 2 μL DNA sample. In order to determine the efficiencies of each plate, samples were run against a standard curve produced by serial diluted reference samples. Only samples that fell within the boundaries of the standard curve were further analyzed. Thermal cycling conditions were as described in the manufacturer’s instructions. Data were collected (Absolute Quantification Method, LightCycler 480 II, Roche, Germany), and samples were assayed using triplicate for each gene of interest. Results were normalized to the expression of the housekeeping genes, *HPRT* and *36B4*.

### Statistical analysis

All results are displayed as means ± S.E. One-way ANOVA was used for evaluation of the differences, applying the Wilcoxon test. *P* < 0.05 was considered significant. JMP and GraphPad Prism 8 software were used for calculation and drawing of the results. All experiments were performed at least three times with at least three replicates.

## RESULTS

A sample of 517 individuals, were analyzed by Affymetrix (unpublished result). A list of candidate CNVs was generated from the Affymetrix microarray GeneChip^®^ miRNA Array results. A list of candidate CNVs were selected to follow up in the pregnant mice model. Remarkably, chromosomes 2, 3, 5, 11, 16, and 17 contained more genes undergoing frequent copy number gain in humans, whereas a high frequency of copy number losses were observed on chromosomes 16 and 21. These human CNVs were aligned to the mouse CNV loci (via the UCSC LiftOver tool available at https://genome.ucsc.edu/cgi-bin/hgLiftOver) summarized in Table 1. Eight CNVs—six gained CNVs (CNV3942, CNV777, CNV357, CNV3153, CNV2343, and CNV1069) and two losses (CNV3188 and CNV3942)—and their corresponding gene expression were selected before, during and after the gestation period. These unique CNVs are mapped to one X-linked and seven autosomal sites as described below (Table1). Possible target genes were selected with copy number-associated changes in gene expression levels.

**Table 1:** CNVs homolog in mouse model.

**CNV ID**	**Type**	**CNV size (bp)**	**Coordinates**	**Size (bp)**	**Gene**	**Liver tissue/Blood samples**
**CNV3153**	Gain	540	chr11:32221443-32221972	540	*RHBDF1*	+/+
**CNV357**	Gain	7132	chr5:113575442-113575601	7132	*WSCD2*	+/+
**CNV3942**	Loss	2134	chr10:76541486-76542556	2134	*LSS*	+/+
**CNV2343**	Gain	3965	chr7:141627749-141630493	3965	*AP2A2*	+/+
**CNV777**	Gain	584	chr16:29229859-29229989	584	*PLAAT1*	+/+
**CNV1069**	Gain	22478	chr13:73784492-73795314	22478	*SLC12A7*	+/+
**CNV3510**	Gain	1400	chrX:68319572-68319799	1400	*GM3951*	+/−
**CNV3188**	Loss	107843	chr16:13724993-13729418	107843	*PLA2G10*	+/+

### DNA copy number changes in liver and blood tissues

**Figure 2:**
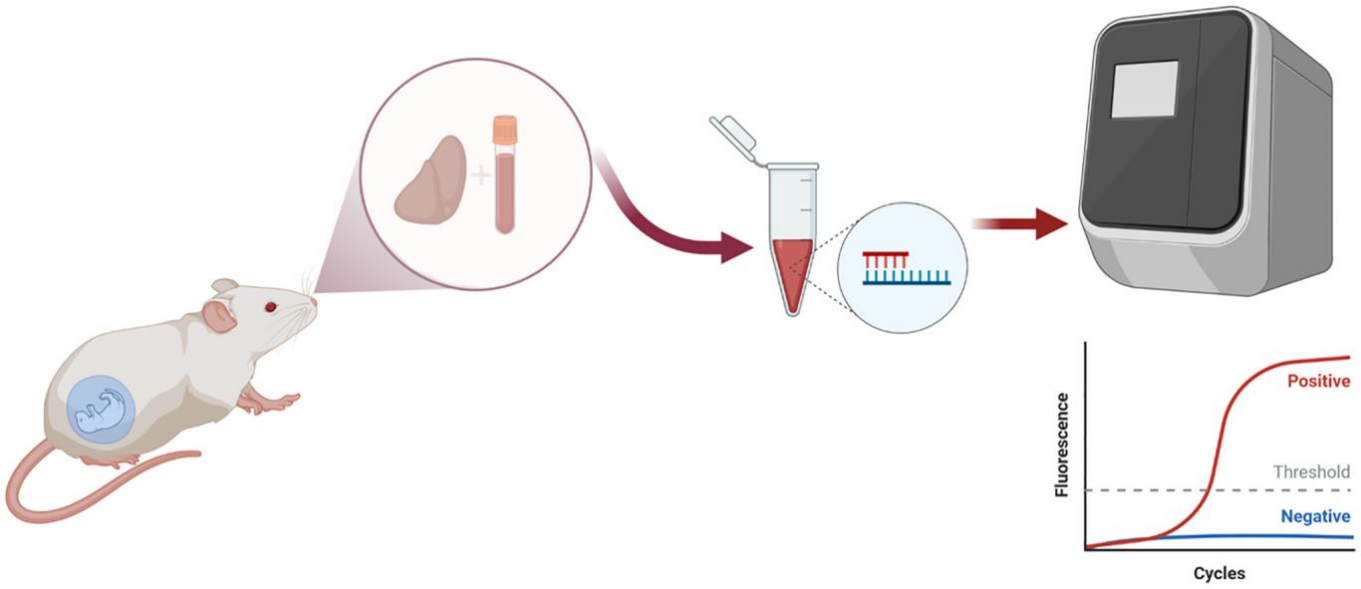
Schematic diagram of the DNA and RNA extraction from tissue liver and DNA extraction from blood samples.

**Figure 3:**
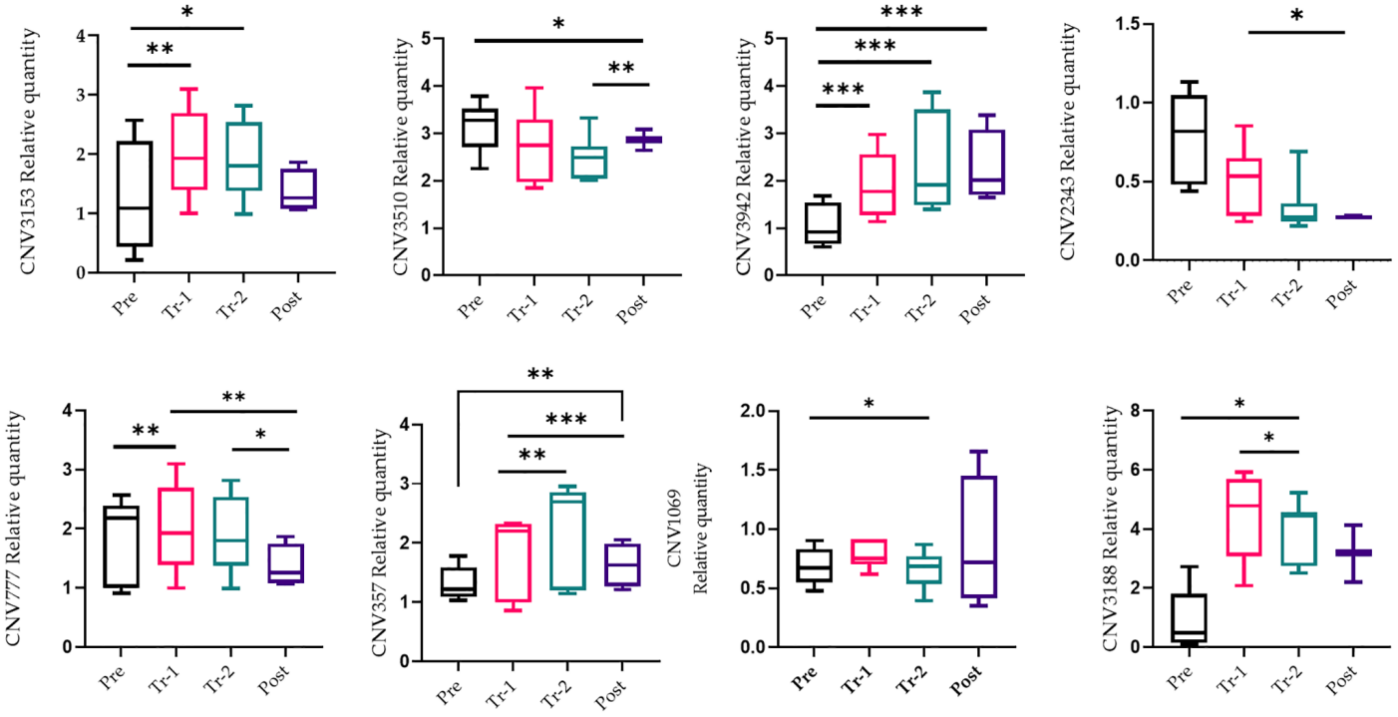
**qRT-PCR confirmation of the copy number variations (CNV).** qRT-PCR analysis on liver tissue was performed during pregnancy (pre-pregnancy, first trimester, second trimester, and after delivery). All results were presented as averages of three independent experiments with three repeats each. Statistical significance between groups is determined by the Wilcoxon test. ^*^p < 0.05, ^*^p < 0.01. Abbreviation: NS: not significant.

**Figure 4:**
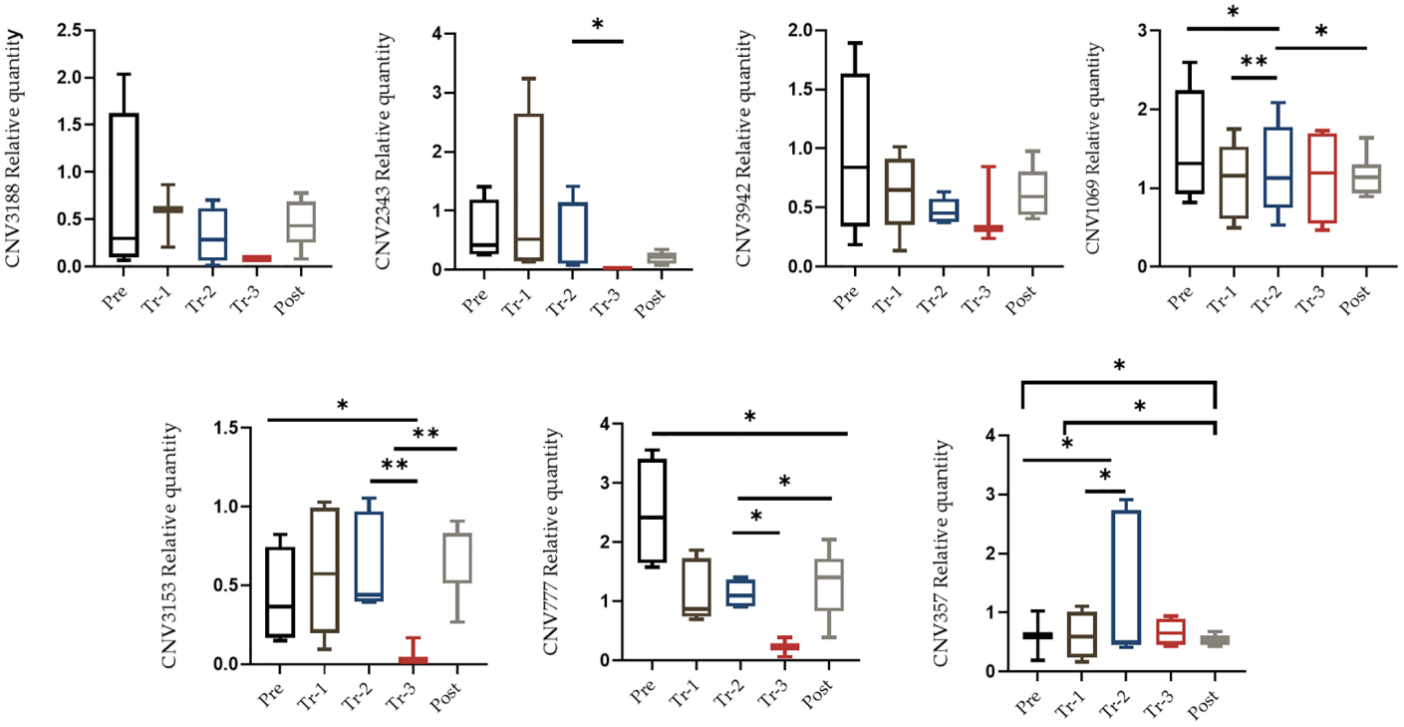
**qRT-PCR confirmation of the CNV.** qRT-PCR analysis was performed on blood samples in order to quantify candidate CNV. qRT-PCR analysis on blood was performed during pregnancy (pre-pregnancy, first trimester, second trimester, third trimester and after delivery). All results were presented as averages of three independent experiments with three repeats each (mean ± S.E). Statistical significance between groups determined by one-way ANOVA or Wilcoxon test. ^*^p value < 0.05, ^**^p value < 0.01, ^***^p value < 0.005.

Twenty-eight liver tissues were obtained from four stages (baseline, first and second trimesters, and post-delivery) of the mice pregnancies (a gift from Prof. Rajat Singh at David Geffen School of Medicine at UCLA). DNA was isolated from these tissues and qRT-PCR was applied to quantify copy number changes. Selected qRT-PCR primers were design for reporter CNVs located in the regions, and a comparison was made between the CNVs at multiple stages of pregnancy. Figure 2 illustrates each CNV qRT-PCR and its alteration analysis (gain or loss) in each phase when normalized to housekeeping gene 36B4. We searched for a pattern during pregnancy in which there was either a gain or a loss of CNVs as the pregnancy progressed, and a change of direction shortly after giving birth. There was a statistically significant difference in the number of gains and losses in the cumulative span of all CNVs in the liver and blood tissue sample (Figures 3 and 4, Supplementary Table 3). For the most part, tissue samples exhibited trend toward an increase in the number of CNVs in the first trimester compared to the second trimester of pregnancies, and a loss in the number of CNVs after delivery, except CNV1069, where we showed a gain in copy number post-delivery (*P* < 0.05, Figure 3; Supplementary Table 3). When comparing the blood cases in the first trimester, there was a mix results in copy number variation; part of the copy number was gained, and another part was lost (Figure 4; Supplementary Table 3). The main difference in the CNV was in the third trimester, as we expected. We perceived a significant loss of CNV (90%) in the third trimester followed by a gain after delivery. Many studies have reported that the high load of CNVs in pregnancy arises mainly from somatic duplications. They have established that the placenta, from late pregnancy complications like preeclampsia, gestational diabetes, and fetal growth disturbances, showed a significantly lower number of CNVs [13, 14].

### Copy number-related gene expression in mice liver tissue

We next assessed the gene’s function that are in close proximity to the candidate CNV loci. qRT-PCR was used to determine the expression pattern of six selected candidate genes (*APA2A, WSCD2, LSS, RBDHF1, PLAAT1*, and *SCL17A2*) in 28 liver tissues.

**Figure 5:**
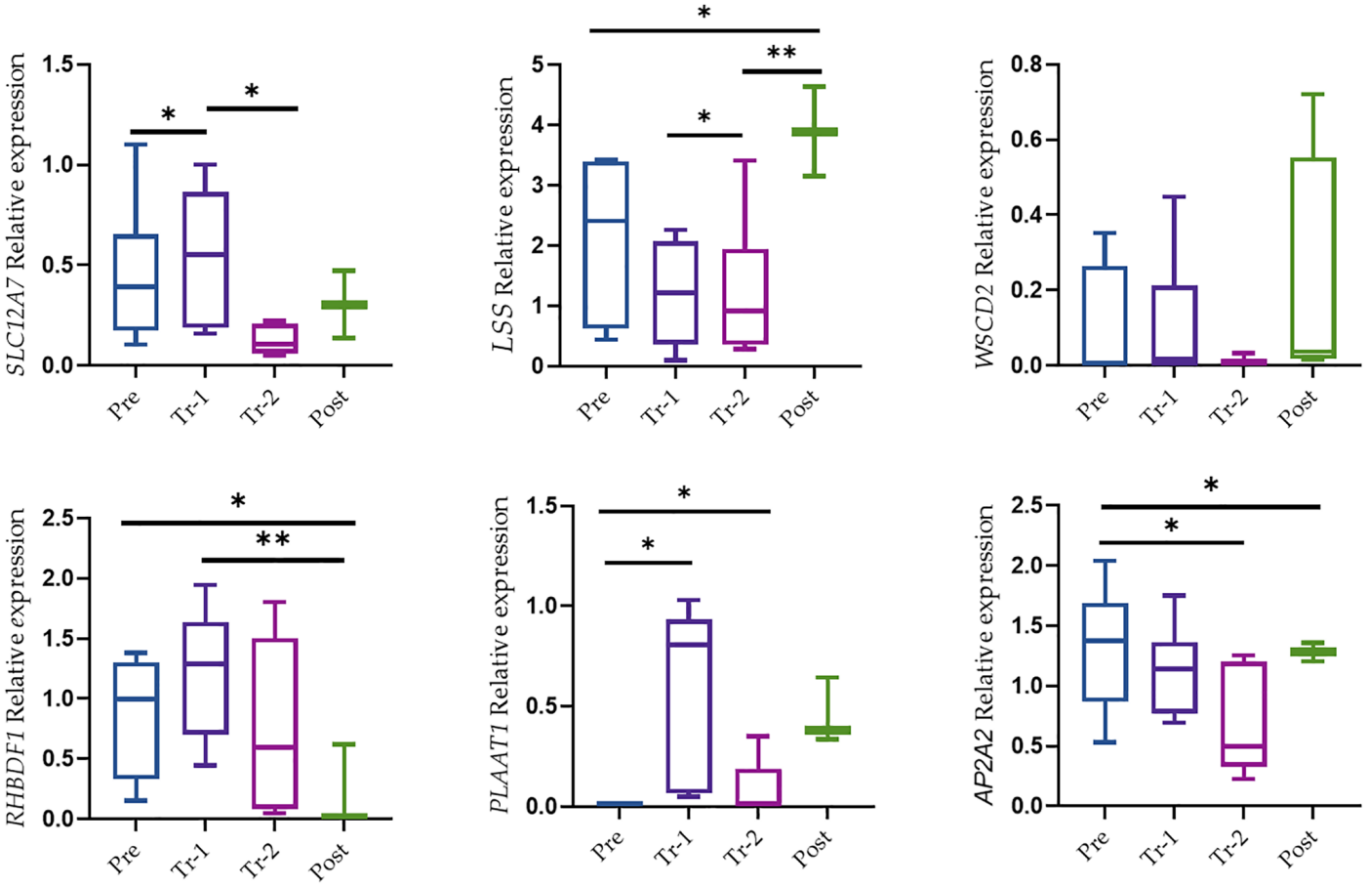
**The mRNA level of genes in liver tissue.** Relative expression was from expression qRT-PCR. Data were presented as Box and Whisker plots with ^*^P < 0.05, ^**^P < 0.005, and ^***^P < 0.0005. All results were presented as averages of three independent experiments with three repeats each (mean ± S.E). Statistical significances between groups were determined by one-way AN OVA or Wilcoxon test.

Relative expression of genes in the liver tissue of the gestation period measured by qRT–PCR is presented in Figure 5. These CNVs ranged in size from 540 kb to 107843 kb, yet their gene content was low. CNV2343 gains 3,965 kb on Chr7 encompass the *AP2A2* gene. *AP2A2* gene function may be related to several mechanisms including pathogenesis like Alzheimer's disease (Nelson et al., 2021; Taneera et al., 2014). We found decreased *AP2A2* gene expression in trimester 2 followed by an increase after labor (*p* < 0.05).

The CNV of 22,478 on Chr13 appears to be in close proximity to the *SCL17A2* gene. *SCL12A7* gene expression was the only one that showed a relatively low expression level in trimester 2 (*p* < 0.05) followed by an increase in gene expression after labor, although this increase was not significant. CNV3153 on Chr11 gains 540 kb overlapped the *RHBDF1* gene. *RHBDF1* gene over expression may have a role in tumorigenesis and prompted us to investigate the role of *RHBDF1* in the early stages of cancer development. However, it is important to note that the expression level of *RHBDF1* after labor was low (*p* < 0.005). *LSS* gene expression showed gradually decreased expression across the gestation period following an increase in gene expression after labor (*p* < 0.05), while *PLAAT1* showed a significant increase in trimester 1 followed by a decrease in trimester 2 (*p* < 0.05). A loss of CNV of 7132 on Chr5 that contains the *WSCD2* gene showed no significant changes in gene expression.

Statistics of the DNA copy numbers and candidate gene expression during gestation are presented in Supplementary Table 1.

## DISCUSSION

The primary aim of this study was to establish pregnancy as a model for aging *in vivo*. We demonstrated that candidate CNVs associated with longevity in human (unpublished results) are correlated with gene expression changes in multiple stages of gestation (each gestational period represents a decade of aging) and are involved in the rejuvenation that occurs soon after labor.

CNV is a type of genetic variation that contributes to phenotypic changes in mammals, either directly affecting disease or part of its development [15, 16]. The analysis of genes affected by rare CNVs has confirmed the crucial role of abnormalities in synapse formation and maintenance, but has also identified other affected pathways, including cellular proliferation and motility, GTPase/Ras signaling, and neurogenesis. Our study applied unique CNVs that were found to be associated with longevity to investigate their role in chromosomal gain or loss in pregnancy. We aimed to evaluate a similar genetic predisposition between longevity and pregnancy by examining the same CNVs to test whether genetic markers for physical degradation as a part of screening in gestation also help establish a potential model for longevity. This study was designed to test the hypothesis that pregnancy may serve as a model for aging and is associated with several molecular changes such as altered metabolic changes, regulated gene expression, and accumulated DNA damage. These metabolic changes impact maintenance mechanisms and pathway regulation on the cellular level, leading to physiological imbalances and pathologies. In addition, CNVs are a notable reason of genomic disorders with Mendelian inheritance and could also contribute to complex diseases with multifactorial etiology (Finding the missing heritability of complex diseases.). We assumed a change in DNA copy number during the gestation period and recovery afterward. We tested eight significant CNVs associated with interest or overlapped genes for validation using qRT-PCR. Evaluating liver tissue and blood from the gestation period with the same CNVs associated with longevity can shed light on the range of phenotypes related to CNVs losses or gains.

CNV can show more extreme impacts on gene expression and function, for instance by changing gene dosage, disrupting coding sequences, or perturbing long-range gene regulation. Additionally, gene expression is a crucial intermediate phenotype, and therefore genetic variations such as CNV linked to gene expression can cause the genetic basis of higher-order traits like disease risk.

We detected six genes that overlapped with the longevity associated CNVs: *WSCD2, PLAAT1, RHBDF1, APA2A, LSS*, and *SCL12A7*. Interestingly, CNV or single nucleotide changes located within enhancers and miRNAs may affect gene expression levels during development. *AP2A2* is a component of an evolutionarily conserved adaptor protein complex; AP-2 is expressed robustly in many human tissues, including in the brain [17]. The AP-2 complex interacts directly with lipids and its function can link with multiple mechanisms linked to Alzheimer's disease pathogenesis, bronchitis, and chronic obstructive pulmonary disease [18, 19]. As we mentioned before many pathologies exhibit similar in pregnancy and elderly. In the present study, CNV2343 is overlapping with the *AP2A2* gene. We found a significant gradual decline in *AP2A2* expression in trimesters 1 and 2 followed by increases in the expression level after labor. These results are in accordance with our predictions that physiological and cellular degradation is seen in the pregnant body and gradual recovery of pre-partum functions occurs. Such performance is in line with reported physiological degradation during aging [3].

The *WSCD2* gene has been found to be associated with insulin sensitivity [20]. The *WSCD2* gene is involved in glucose metabolism in human islet cell expression studies and overlapped with CNV357 [21]. In addition, *WSCD2* was found to interact with mtSNP in the mitochondrially-encoded NADH dehydrogenase 4 (MT-ND4) gene. Bushel et al. suggested that low expression of the *WSCD2* gene coupled with large nuclei is associated with an increased risk of death and mortality [22]. However, *WSCD2* has no known function and there is insufficient biological information to theorize a likely mechanism of how *WSCD2* gene expression can play a role in the effect of nuclei size or how the mito-nuclear interaction contributes to the pathogenesis of breast cancer [23]. In our study, we found increased expression level which was not significant. Another significant CNV (CNV3942) associated with the LSS gene that encodes lanosterol synthase enzyme which converts (S)-2,3-oxidosqualene to lanosterol in the cholesterol biosynthesis pathway and which has the crucial function of the metabolic pathway in the homeostasis of hair growth and disruption of expression level caused congenital cataracts and skin lesions. It was also compelling that mutation in the *LSS* gene affects vitamin D metabolism and cholesterol metabolism. Our study demonstrated gradual decrease followed by an increase after labor. *PLAAT1* belongs to the phospholipase A/acyltransferase (*PLAAT*) family and acts as Ca2+-independent NAT, overexpressed in skeletal muscle, the brain, and heart in both human and rodent. Pu et al. observed that *PLAAT1* was found to create an immune-related prognostic model [24]. In our study, we found a decrease in *PLAAT1* expression level that overlapped with CNV777 during trimester 2 followed by an increase in expression after labor, although this did not reach statistical significance.

*SCL12A7* was also shown to promote tumor cell growth *in vitro* and *in vivo*. Previous studies showed that *SLC12A7* copy gains may serve as a putative molecular indicator of malignancy in indeterminate adrenal tumors, including a marker of malfunctioning tumors [25, 26]. *SLC12A7* gene expression levels have been shown to be significantly overexpressed during trimester 1 followed by decreased expression levels during trimester 2. These results are in accordance with our predictions but contrast with the results obtained when testing the *RHBDF1* gene. The *RHBDF1* gene shows an association with arsenic-induced skin lesions and is essential to epithelial cancer cell growth and highly expressed in breast cancer [26, 27]. Although the function of RHBDF1 in humans is unknown, it serves a variety of important functions in other organisms. *RHBDF1* has been implicated in neurological disorders including Alzheimer’s and Parkinson’s diseases, in addition to cancer, inflammation, and skin diseases [3, 28–30]. However, CNV is disease-affecting or does not depend on various factors, like gene content—for example, a CNV that is gene-rich is more likely to affect a phenotype than one having only a few or no genes. Understanding the effect of CNV on pregnancy complications allows medical intervention and is essential for experimental treatment. We have shown the similarity between pregnancy and the aging process that has previously been reported [3, 31], assuming that in pregnancy there is an extraordinary regeneration that returns the damage caused by stress to the original levels after labor compared to aging, which is typified by direct degradation without recovery. The damage resolves after pregnancy with minimal residual effects and occurs up to a few months after birth [30]. Thus, exploring the mechanisms through which such rejuvenation arises may assist with tailoring potential treatment for aging deceleration or even recovery.

In summary, pregnancy as an enhanced and effective aging model will expand our understanding of the complex characteristics of a healthy lifespan. While postpartum recovery contributes to the completion of reversal mechanisms, these mechanisms may translate to aging therapy and might delay the aging phenotype as well as reverse aging processes.

## Supplementary Materials

Supplementary Tables
