# Supplementary material for: Copy number variation as a tool for implementing pregnancy as an aging model
Source: Aging (Albany NY). 2023 Aug 28;15(16):7922–32. doi: 10.18632/aging.204936 (PMC10496986; doi:10.18632/aging.204936)
Supplement: Supplementary Tables [file aging-15-204936-s001.pdf]

# SUPPLEMENTARY TABLES

## Supplementary Table 1. List of primers.

| Primer | Forward sequence | Reverse sequence |
|---|---|---|
| **CNV3153** | CAGCTCTACTAGCGTGCTCC | AGGTGGCGTCTGAAACTCTG |
| **CNV357** | AACCGCAAATATGGAGGCCA | ATTACCCGCACCTCACACTG |
| **CNV2343** | GCAAGTGAGAGGGGTGAACT | TTGCAGGATTCGGGTCAACT |
| **CNV777** | TGAGCAGGCGATTTGTGTGT | TCACGTAGCCATCACCCAAG |
| **CNV3942** | GTTGGCTGCCCATAGGTTCT | CCAGCCACCACTTAACCACT |
| **CNV3510** | GCTTCTTGGCCACCTTTTCG | GCTCGCCATCCACTTCCTAA |
| **CNV3188** | TTAGAGGCACTGGCCCAAGA | CTGCCAATGTACTCCACCGT |
| **CNV1069** | AGAGCACATGTCATGGTGTGA | GAGAGGGACAGCTCTACAGG |

## Supplementary Table 2. List of gene primers.

| Gene | CNV name | Forward sequence | Reverse sequence |
|---|---|---|---|
| ***RHBDF1*** | CNV3153 | TCCTCTGCATACTGGAGCCT | CAGTGAGACAAGCCTGGGAG |
| ***WSCD2*** | CNV357 | GGGCCTTGGATCACACTCTC | GGGCCTTGGATCACACTCTC |
| ***APA2A*** | CNV2343 | GATGGAGTCACCCAGGGTCT | TGCTCAAGCTGGGTTGTCTT |
| ***SLC12A7*** | CNV1069 | GTTCCGACGCCAACATGTC | CGGCAGTAGCGCAGAATGG |
| ***PLA2G10*** | CNV3188 | CACCAAGGCTCCAGCTCTTT | GGCCCACACATAGGAGAGTG |
| ***LSS*** | CNV3942 | TCAGGTAAAGGGCTGGCAAG | TGTGTCCAGTCCCAAAGAGTG |

## Supplementary Table 3. Relationship between DNA copy numbers/candidate gene expression during gestation.

| Tissue/ Blood | Gestation period | CNV/ Gene | ***P* value** | | | | | | | | | |
|---|---|---|---|---|---|---|---|---|---|---|---|---|
| | | | **Pre – Tr-1** | **Pre – Tr-2** | **Pre – Tr-3** | **Pre – Post** | **Tr-1 – Tr-2** | **Tr-1 – Tr-3** | **Tr-1 – Post** | **Tr-2 – Post** | **Tr-2 – Tr-3** | **Tr-3 – Post** |
| **Tissue** | Pre – Tr-1 | CNV3153 | **0.0393** | 0.0044 | | **0.5471** | 0.4089 | | 0.2897 | 0.0873 | | |
| | | CNV3942 | **0.0004** | **0.0008** | | **0.0002** | 0.6624 | | 0.05368 | 0.3051 | | |
| | | CNV777 | **0.0196** | 0.3793 | | 0.2801 | 0.0916 | | **0.0012** | **0.0486** | | |
| | | CNV357 | 0.3096 | 0.0711 | | **0.0091** | **0.0066** | | **0.0009** | 0.2406 | | |
| | | CNV3188 | 0.7695 | 0.0371 | | 0.07344 | **0.0233** | | 0.568 | 0.1654 | | |
| | | CNV3510 | 0.986 | 0.3761 | | **0.0459** | 0.3885 | | 0.0548 | **0.0076** | | |
| | | CNV2343 | 0.2706 | 0.9449 | | 0.0909 | 0.2295 | | **0.0064** | 0.0733 | | |
| | | CNV1069 | 0.2355 | **0.0392** | | 0.411 | 0.3702 | | 0.8805 | 0.377 | | |
| **Blood** | Pre – Tr-1 | CNV3153 | 0.2163 | 0.9177 | **0.0185** | 0.9276 | 0.14 | 0.19 | 0.133 | 0.9863 | **0.0083** | **0.0073** |
| | | CNV3942 | 0.7785 | 0.6098 | 0.1539 | 0.7907 | 0.8123 | 0.2119 | 0.9713 | 0.7689 | 0.2781 | 0.1794 |
| | | CNV777 | 0.1765 | 0.984 | 0.0594 | **0.0146** | **0.1493** | 0.5223 | 0.2468 | **0.0085** | 0.0449 | **0.6584** |
| | | CNV357 | 0.6658 | **0.0341** | 0.2279 | **0.0396** | **0.0375** | 0.3404 | **0.0435** | 0.8818 | 0.2867 | 0.3351 |
| | | CNV3188 | 0.9156 | **0.205** | **0.6854** | 0.304 | 0.158 | 0.534 | 0.2398 | 0.7325 | 0.1125 | 0.1677 |
| | | CNV2343 | 0.47 | 0.1284 | 0.4423 | 0.2405 | 0.434 | 0.1662 | 0.6936 | 0.6519 | 0.0394 | **0.0742** |
| | | CNV1069 | 0.5316 | **0.341** | 0.6323 | 0.7696 | **0.0045** | 0.2642 | 0.69 | **0.0067** | 0.106 | **0.4093** |
| **Gene expression** | Pre – Tr-1 | SLC12A7 | **0.0331** | 0.753 | | 0.0779 | 0.0193 | | 0.9029 | 0.0508 | | |
| | | LSS | 0.1078 | 0.9386 | | 0.0148 | 0.0296 | | 0.1968 | 0.0026 | | |
| | | WCSD2 | 0.4892 | 0.94 | | 0.5643 | 0.5444 | | 0.9995 | 0.6121 | | |
| | | RHBDF1 | 0.2494 | 0.4785 | | **0.0219** | 0.0952 | | **0.0027** | 0.1092 | | |
| | | PLAAT1 | **0.0177** | **0.0679** | | 0.6326 | 0.6347 | | 0.1211 | 0.2681 | | |
| | | AP2A2 | 0.4298 | **0.0182** | | **0.0452** | 0.1146 | | 0.132 | 0.6124 | | |
